# Altered sucrose synthase and invertase expression affects the local and systemic sugar metabolism of nematode-infected *Arabidopsis thaliana* plants

**DOI:** 10.1093/jxb/ert359

**Published:** 2013-11-01

**Authors:** Susana Cabello, Cindy Lorenz, Sara Crespo, Javier Cabrera, Roland Ludwig, Carolina Escobar, Julia Hofmann

**Affiliations:** ^1^University of Natural Resources and Applied Life Sciences, Department of Crop Sciences, Konrad Lorenz Str. 24, Tulln a. d. Donau A-3430, Austria; ^2^University of Natural Resources and Applied Life Sciences, Department of Food Sciences and Technology, Vienna, Austria; ^3^Universidad de Castilla-La Mancha, Facultad de Ciencias del Medio Ambiente, Avenida de Carlos III s/n, 45071 Toledo, Spain

**Keywords:** Cytosolic invertase, enzyme activity, *Heterodera schachtii*, *Meloidogyne javanica*, nematode, neutral invertase, plant pathogen, sucrose synthase.

## Abstract

Sedentary endoparasitic nematodes of plants induce highly specific feeding cells in the root central cylinder. From these, the obligate parasites withdraw all required nutrients. The feeding cells were described as sink tissues in the plant’s circulation system that are supplied with phloem-derived solutes such as sugars. Currently, there are several publications describing mechanisms of sugar import into the feeding cells. However, sugar processing has not been studied so far. Thus, in the present work, the roles of the sucrose-cleaving enzymes sucrose synthases (SUS) and invertases (INV) in the development of *Heterodera schachtii* were studied. Gene expression analyses indicate that both enzymes are regulated transcriptionally. Nematode development was enhanced on multiple *INV* and *SUS* mutants. Syncytia of these mutants were characterized by altered enzyme activity and changing sugar pool sizes. Further, the analyses revealed systemically affected sugar levels and enzyme activities in the shoots of the tested mutants, suggesting changes in the source–sink relationship. Finally, the development of the root-knot nematode *Meloidogyne javanica* was studied in different *INV* and *SUS* mutants and wild-type *Arabidopsis* plants. Similar effects on the development of both sedentary endoparasitic nematode species (root-knot and cyst nematode) were observed, suggesting a more general role of sucrose-degrading enzymes during plant–nematode interactions.

## Abstract (precis)

Changes in the gene expression of sucrose synthases and invertases affected the local and systemic plant metabolism and communication, source–sink relationships, thus nutrition and development of sedentary endo-parasitic nematodes

## Introduction

Sedentary endoparasitic nematodes such as cyst and root-knot nematodes induce highly specific feeding cell systems in plant roots. The cyst nematode *Heterodera schachtii* infects different Chenopodiaceae and Brassicaceae species. Subsequently, second stage juveniles (J2s) invade host roots and migrate intracellularly towards the vasculature where they pierce a single cell with their stylets to inject nematode saliva. This provokes a dramatic morphological and physiological re-organization, as well as cell wall openings along plasmodesmata, resulting in the formation of highly specialized syncytial feeding cells (Golinowski *et al.*, 1996). J2s of the root-knot nematodes migrate intercellularly towards the root tip where they pass the endodermis eventually to reach the vasculature. The injection of nematode secretions leads to the formation of several giant cells that emerge by successive karyokinesis events without cytokinesis (Huang and Maggenti, 1969). In the following days, syncytia and giant cells emerge as strong sink tissues that serve the endoparasitic nematodes as the sole source of energy, nutrients, and water (McClure, 1977; Böckenhoff *et al.*, 1996).

Sucrose can be assumed to be the major source of carbohydrate input into nematode-induced feeding sites (NFS) in *Arabidopsis thaliana* roots. First, sucrose was described as the main transported sugar in the phloem of this plant species (Haritatos *et al.*, 2000). Secondly, metabolite analyses revealed significantly increased sucrose levels in syncytia and giant cells (Hofmann *et al.*, 2007; Baldacci-Cresp *et al.*, 2011). While sugar import mechanisms have been studied intensively in recent years (Juergensen *et al.*, 2003; Hammes *et al.*, 2005; Hoth *et al.*, 2005; Hofmann *et al.*, 2007, 2009, 2010b) there is little information about their processing in NFS.

In sink tissues, sucrose is cleaved to make glucose and fructose available for energy-gaining reactions, macromolecule and amino acid biosynthesis (Ehness *et al.*, 1997). This reaction is performed by two enzyme families, invertases (INVs) and sucrose synthases (SUSs) (Koch, 2004; Vargas and Salerno, 2010). Both use sucrose as a substrate but deliver different products. INVs break sucrose irreversibly into glucose and fructose, and SUSs produce fructose and UDP-glucose, enabling reversible sucrose synthesis.

The *A. thaliana* genome encodes six *SUS* genes (Baud *et al.*, 2004; Bieniawska *et al.*, 2007; Barratt *et al.*, 2009) with specific temporal and spatial expression patterns (Bieniawska *et al.*, 2007; Angeles-Nunez and Tiessen, 2010). Proteins for the first four can be found in the soluble extract of mature plants, whereas the remaining isoforms are found in the non-soluble fraction of roots and stems (*AtSUS5*) and in the hypocotyl (*AtSUS6*) (Barratt *et al.*, 2009). In *A. thaliana*, single *SUS* T-DNA insertion lines showed no changes in sugar composition, while multiple mutants did (Bieniawska *et al.*, 2007). However, all lines showed no phenotypic effects. Thus, so far there are only speculations about the role of different SUS isoforms.

INVs are grouped based on their pH optimum. Acidic INVs (pH 4.5–5.0) are active along cell walls (CWINVs) and in vacuoles (VINVs). In *A. thaliana*, six and two genes code for CWINVs and VINVs, respectively. Neutral or alkaline INVs (A/N-INVs) that are also named cytosolic invertases (CINVs) are active between pH 6.5 and 8.0. They are coded for by nine different genes in *A. thaliana* that are expressed in the cytosol as well as in the plasma membrane, the nucleus, chloroplasts, and mitochondria (reviewed by Vargas and Salerno, 2010). The *Atcinv1/Atcinv2* double T-DNA insertion line showed reduced root growth and abnormal cell division, indicating the importance of *AtCINV1* and *AtCINV2* for plant growth and development (Barratt *et al.*, 2009; Vargas and Salerno, 2010).

In additrion to their importance for plant development, the expression of *SUS* and *INV* genes was reported to be regulated under abiotic and biotic stresses. *INV* genes were described to respond to high salinity, drought, or anaerobiosis, as well as to low temperature or hormone treatment (Marana *et al.*, 1990; Zeng *et al.*, 1999; Baud *et al.*, 2004). Further, *SUS* transcript levels increased during *Glomus intraradices* colonization of *Phaseolus vulgaris* (Blee and Anderson, 2002), the formation of root nodules in *Pisum sativum* (Gordon *et al.*, 1999), the interaction between the symbiont *Sinorhizobium meliloti* and its host *Medicago truncatula* (Ferrarini *et al.*, 2008), and in different *Vitis vinifera* varieties undergoing phytoplasma infection (Hren *et al.*, 2009). Currently, there is no information about the role of A/N-INVs during plant–pathogen interaction. Previous transcriptome analyses on NFS showed that the the currently described 17 INV and six SUS isoforms were either up-, down-, or not regulated as compared with the control (Szakasits *et al.*, 2009; Barcala *et al.*, 2010).

In order to study sucrose breakdown in cyst nematode-infected plants, gene expression, and metabolite analyses, enzyme activity assays and nematode infection and development tests were performed in single and multiple *INV* and *SUS* mutants compared with the wild type. Further, the effect of *H. schachtii* root infection on systemic sucrose processing was studied in selected mutants. Infection tests were also performed on the same T-DNA lines with *Meloidogyne javanica*. The results showed that nematode infection affects local and systemic sucrose processing in plants and that changes in plant sucrose processing affect nematode development.

## Materials and methods

### Plant growth and nematode inoculation

Sterile *Arabidopsis* (mutants and wild-type Col-0) seeds were sown on Knop medium-containing Petri dishes under axenic conditions. To avoid sucrose supplement on the roots that would falsify the results of the analyses, two types of Knop medium were prepared. Each Petri dish was one-third filled with Knop medium supplemented with sucrose. In this area, the seeds germinated so that the hypocotyl and the first millimetres of the main root were provided with sucrose, enabling successful plant growth. The other two-thirds of the dish contained Knop medium without any sugar added so that the collected root and syncytium samples were free of sucrose. For *M. javanica* (Portillo *et al.*, 2009) infection, plants were grown as described but in Gamborg medium. All plants were cultivated under 16h light/8h dark, at 25 °C. Twelve-day-old plants were inoculated with ~50 freshly hatched sterile *H. schachtii* J2s per plant obtained from a sterile stock culture (Sijmons *et al.*, 1991) or with 40 freshly hatched *M. javanica* J2s multiplied *in vitro* on cucumber roots (Cucumissativus cv. Hoffmanns Giganta).

Syncytia, shoots from infected plants (i-shoots), and roots and shoots from control plants were collected at 5, 10, and 15 days after inoculation (dai) in the middle of the photoperiod to avoid diurnal effects and were immediately shock-frozen in liquid nitrogen and stored at –80 °C until use. Samples were pooled from ~30 plants for each replicate and contained ~20mg fresh weight (FW) of material. Samples were collected in 3–5 biologically independent replicates (in total 90–150 plants).

### Mutant selection

Nematode infection tests were performed using the T-DNA single and double mutants listed in Supplementary Table S1 available at *JXB* online. All these lines were used and described in previous publications (Bieniawska *et al.*, 2007; Barratt *et al.*, 2009), providing a solid basis for the current study. The single T-DNA lines were obtained from the Nottingham Arabidopsis Stock Centre (NASC; http://arabidopsis.info) and screened for homozygosity according to the Salk Institute Genomic Analysis Laboratory (http://signal.salk.edu). Multiple T-DNA insertion lines and the *Atsus4* T-DNA insertion line were kindly donated by Dr A. Smith (John Innes Centre, Norwich, UK) (Supplementary Table S1). Primer sequences for testing the mutants are listed in Supplementary Table S2.

### Nematode infection tests

All lines and the wild type were cultivated and inoculated as described above. For *H. schachtii*, females and males 15 dai were counted on 35–40 plants per replicate, resulting in between 140 and 330 plants in total in 4–8 replicates. Results were calculated as the number of nematodes per centimetre of root and finally expressed as the percentage of nematodes developing in the wild type. The female size was measured using an inverted microscope (Axiovert200M, Zeiss, Hallerbergmoos, Germany), studying between five and six female nematodes from five different Petri dishes for each line (25–30 females in total). Female sizes were obtained as μm^2^ and were expressed relative to the size of females that developed on the wild type. Eight weeks after inoculation, 10 brown cysts were collected randomly, crushed open, and suspended in 1ml of gelrite. The number of eggs was counted in 20 aliquots of 10 μl using an Axiovert 200M microscope. All experiments were performed as three biologically independent replicates. For *M. javanica*, the infection tests were performed with 4–5 plates with 10 seeds per plate in two sets of independent experiments per line (a minimum of 80 individual plants per line). Plates were thoroughly examined under the stereomicroscope at 10 dai to determine the number of galls per plant.

### Quantitative reverse transcription–PCR

RNA was extracted from syncytium and control root samples 5, 10, and 15 dai using the RNAeasy QIAGEN extraction kit (Qiagen, Hilden, Germany) including a DNase digest (Qiagen) according to the manufacturer’s protocols. The quality and quantity of RNA were tested with a NanoDrop 2000c (Thermo Scientific, Bremen, Germany) and it was thereafter transcribed into cDNA using SuperScript III reverse transcriptase (Invitrogen, Carlsbad, CA, USA). The quantitative PCR (qPCR) was performed using an ABI PRISM 7300 (Applied Biosystems, Foster City, CA, USA). Each reaction contained 12.5 μl of Platinium SYBER Green qPCR Super Mix (Invitrogen), 2 μl of cDNA, MgCl_2_, and primer according to Supplementary Table S3 at *JXB* online, and water was added to reach a total reaction volume of 25 μl. As internal references, genes coding for *18S* and *UBP22* were used (Hofmann and Grundler, 2007). Samples were tested in three biological replicates, each tested as technical triplicates. Blank samples and dissociation runs were made in order to rule out non-specific amplifications. Results were analysed using the SDS 2.0 software (Applied BioSytems) and were determined by the 2^–ΔΔCt^ method (Schmittgen and Livak, 2008).

### Metabolic analysis

Metabolites from 6–21mg FW of infected tissues of wild-type and T-DNA insertion lines at 15 dai were extracted by methanol/chloroform (Kaplan *et al.*, 2004). From the remaining pellet, starch was digested by α-amylase from *Bacillus licheniformis* (Sigma-Aldrich) and amyloglucosidase (Sigma-Aldrich) to obtain glucose equivalents (Wanek *et al.*, 2001). The polar phase and the glucose equivalents were dried *in vacuo* and stored at –80 °C. Carbohydrates were analysed by high-performance liquid chromatography (HPLC) coupled with pulsed amperometric detection on a Dionex DX500 system using an ED40 electrochemical detector with a gold electrode. Eluents were degassed by flushing with helium. An anion exchange 4×250mm CarboPac PA1 column connected to a 4×50mm guard column was used at 30 °C. The flow rate of the mobile phase was 1ml min^–1^. The initial mobile phase was 20mM NaOH for 20min; then a gradient from 20mM to 200mM NaOH was applied for 10min and the final 200mM NaOH concentration was maintained for a further 10min. Finally, 20mM NaOH was applied for 20min to equilibrate the columns for the next sample (20 μl injection volume). The identification of different carbohydrates was based on commercially available standards (Sigma-Aldrich, Roth, and VWR).

### Enzymatic analysis

Assays for A/N-INVs were modified according to Gibon *et al.* (2004). Proteins were extracted from ~20mg FW of syncytia, i-shoots, and roots and shoots of non-infected plants at 15 dai. Plant material was ground using liquid nitrogen and incubated in extraction buffer according to Gibon *et al.* (2004). The supernatant and pellet from the extract were used to assay the acidic vacuolar and cell wall INV activity, respectively, in acetate/KOH pH 4.5. Neutral INV activity was assayed in the supernate in 50mM HEPES pH 7.0. Both types of reaction were started by adding sucrose to a final concentration of 20mM. The reaction was stopped after 20min by heating for 3min at 95 °C. After stopping the reaction, 40 μl of 0.2M HEPES buffer pH 7 were added to neutralize the pH. Glucose production was detected according to Gibon *et al.* (2004) using a fluorimeter, with an excitation of 571nm and an emission of 585nm (Microplate reader FLUOstar Omega, BMG LABTECH, Offenburg, Germany). Blanks were boiled for 3min before adding the sucrose.

### Statistical analysis

Results are expressed as means± SE, *n*=3–33, all tested in at least three independent biological replicates. Significant differences were calculated using Student’s *t*-test.

## Results

### *Heterodera schachtii* root infection triggers changes in sucrose degradation

**Fig. 1.**
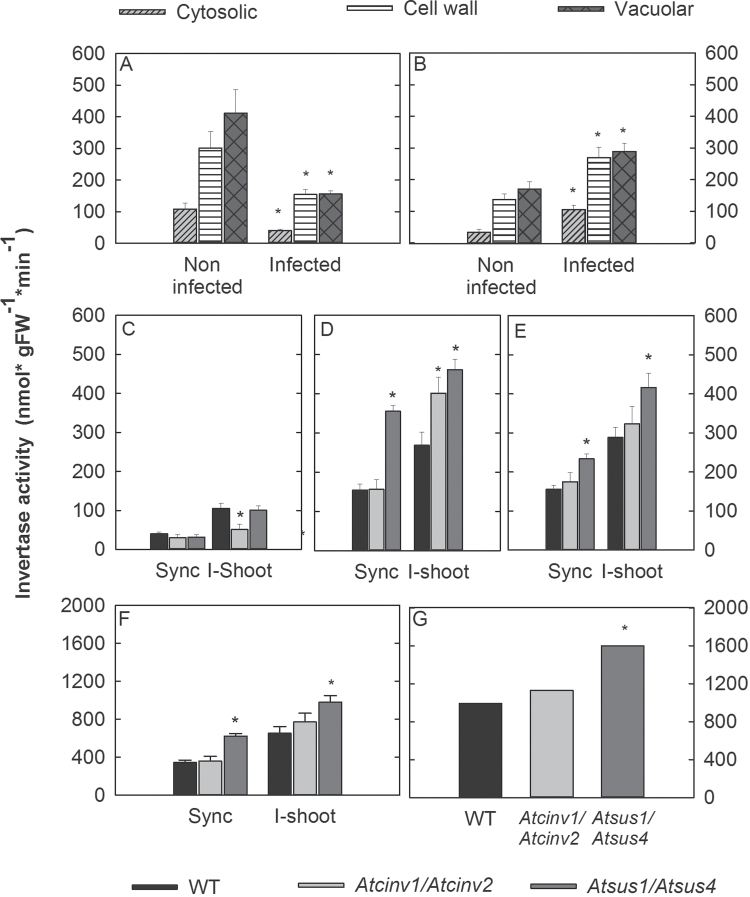
Invertase activity of cytosolic (CINV), cell wall (CWINV), and vacuolar (VINV) invertases in (A) roots and (B) shoots of non-infected and infected plants (15 dai). (C) CINV, (D) CWINV, and (E) VINV activity in syncytia (sync) and shoots of infected plants (i-shoot) of the wild type and *Atcinv1/Atcinv2*, and *Atsus1/Atsus4* T-DNA double mutant lines. Total invertase activity was equivalent to the sum of cytosolic, cell wall, and vacuolar invertases in (F) syncytia and i-shoots, and (G) in whole infected plants. Values are means±SE, *n*=9. * indicates significant differences compared with the non-infected control (Student’s *t*-test, *P*<0.05).

**Fig. 2.**
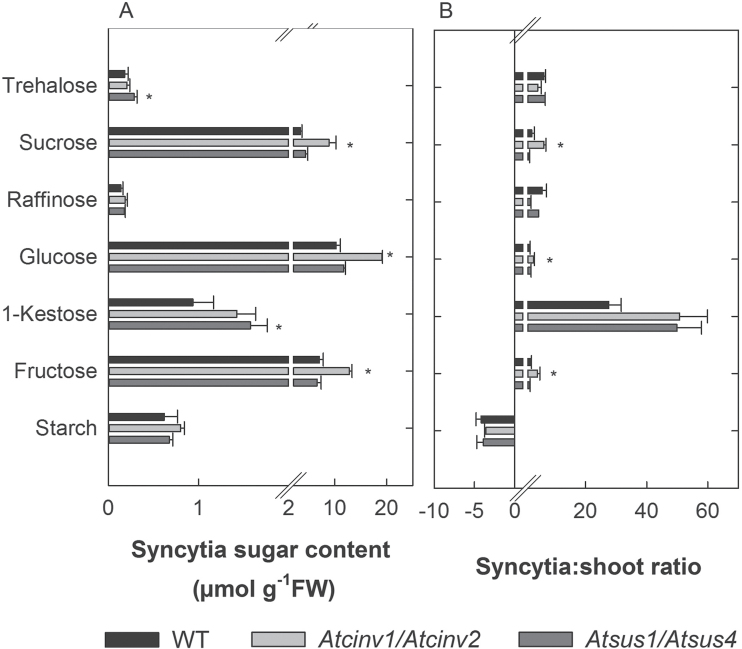
(A) Sugar levels in *H. schachtii*-induced syncytia and (B) syncytia:shoot ratio of sugars in the wild type and *cinv1/cinv2* and *sus1/sus4* T-DNA insertion lines (15 dai). Values are means±SE, *n*=3, * indicates significant differences compared with the wild type (Student’s *t*-test, *P*≤0.05).

Expression levels of 10 *INV* and six *SUS* gene were studied in syncytia at 15 dai compared with control roots using qRT-PCR (Table 1). The analysis showed that *AtVINV1*, *AtCINV1*, *AtCWIN1*, and *AtCWINV6* were significantly down-regulated, and, amongst those, *AtCINV1* showed the strongest changes; *AtSUS1*, *AtSUS4*, and *AtSUS6* were significantly up-regulated. Other members of the *SUS* and *INV* gene families were not differentially expressed (Table 1). The down-regulation of the different *INV* genes was reflected by a decrease in neutral (cytosolic) and acidic (vacuolar and cell wall) INV activity in *H. schachtii*-infected syncytia as compared with the control (Fig. 1A). Further, high levels of glucose and fructose but also sucrose and 1-kestose in nematode-induced syncytia were found (Fig. 2A).

**Table 1.** *Fold change (log*_*2*_ *) expression levels of members of the sucrose synthase (*SUS*) and invertase (*INV*) gene families in* H. schachtii*-induced syncytia (15 dai) analysed by qPCR, and* M. javanica*-induced giant cells and galls (3 dai) analysed by Superamine TeleChem gene chip by* Barcala *et al.* (2010 *) compared with non-infected* A. thaliana *roots*

Locus	Name	*H. schachtii*	*M. javanica* ^*a*^	
		Fold change	Fold change	
		Syncytia	Giant cells	Galls
At1g12240	*AtVINV1*	–1.7±0.6*	0.1	–0.3
At1g62660	*AtVINV2*	–1.0±0.9	–0.5	–0.4
At1g35580	*AtCINV1*	–1.9±0.1***	–0.8	–0.3
At4g09510	*AtCINV2*	0.0±0.7	1.3*	0.5
At3g13790	*AtCWINV1*	–1.8±0.6*	–0.3	0.1
At3g52600	*AtCWINV2*	–0.1±0.4	0.0	–0.1
At1g55120	*AtCWINV3*	0.5±0.2	–0.7	0.9
At2g36190	*AtCWINV4*	–0.6±0.5	–0.4	–0.2
At3g13784	*AtCWINV5*	0.4±0.6	0.0	–0.1
At5g11920	*AtCWINV6*	–1.4±0.3***	–0.3	0.2
At5g20830	*AtSUS1*	2.9±0.3**	2.7*	2.4**
At5g49190	*AtSUS2*	0.5±0.6	–0.1	0.1
At4g02280	*AtSUS3*	0.5±1.6	–1.9	–0.6
At3g43190	*AtSUS4*	3.3±0.1***	–0.1	1.1*
At5g37180	*AtSUS5*	0.7±0.8	–0.3	–0.1
At1g73370	*AtSUS 6*	1.8±0.3*	–0.1	0.1

The neutral invertases At4G34860, At5G22510, At3G06500, At3G05820, At1G56560, At1G72000, and At1G22650, were not analysed during this study.

Values are means±SE, *n*=3, (Student’s *t*-test, *P<0.05; **P<0.01; ***P<0.001).

^*a*^ Data published in Barcala *et al.* (2010).

### Lack of *INV* and *SUS* expression affects *H. schachtii* development and offspring production

**Fig. 3.**
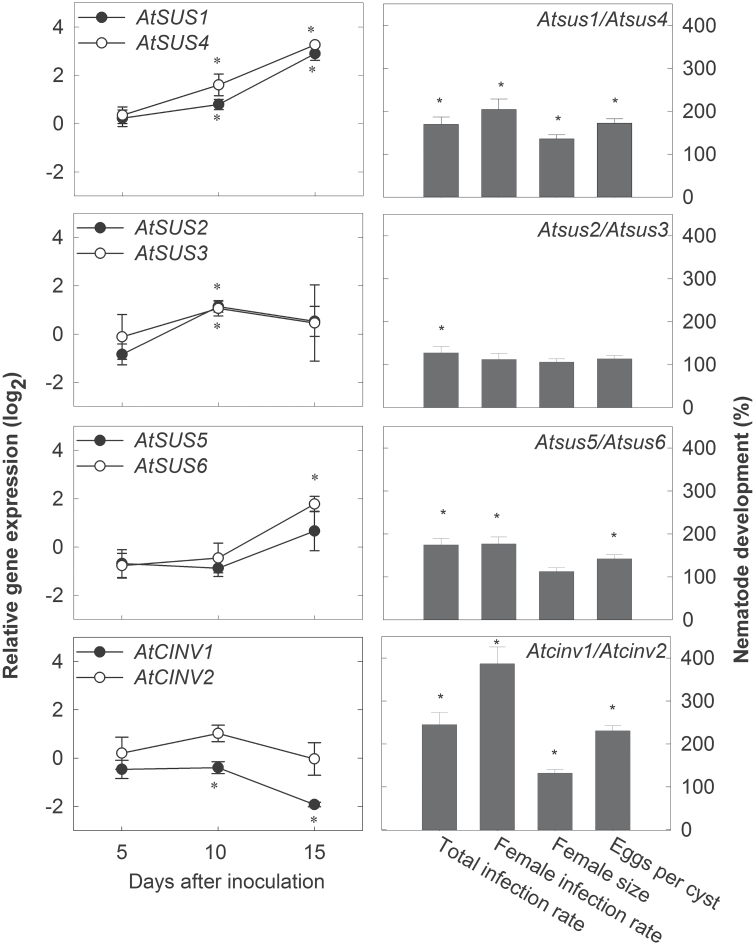
Fold change (log_2_) expression levels of *SUS* and *CINV* genes in *H. schachtii*-induced syncytia compared with non-infected *A. thaliana* roots. Values are means±SE, *n*=3 (left-hand side). Total and female nematode infection rates, female size, and eggs per cyst of *H. schachtii* on multiple *A. thaliana sus* and *cinv* T-DNA double mutant lines relative to the wild type. Values are means±SE, *n*=14–33 (right-hand side). * indicates significant differences (Student’s *t*-test, *P*≤0.05).

**Fig. 4.**
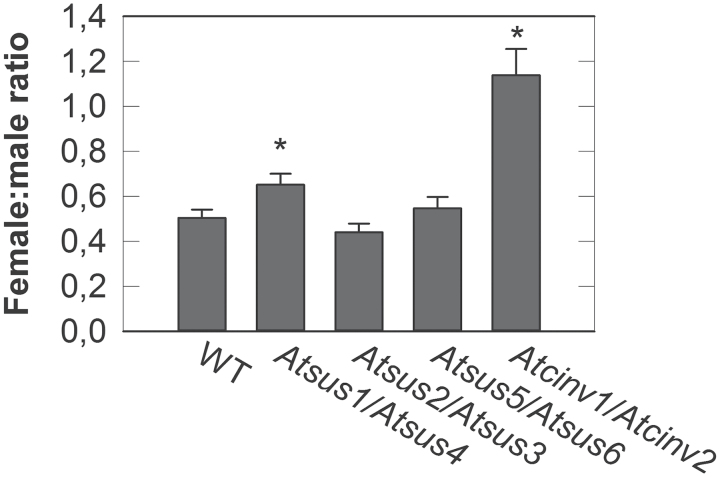
Female:male ratio of *H. schachtii* developing on the wild type and on multiple *A. thaliana SUS* and *CINV* T-DNA insertion lines. Values are means±SE, *n*=14–33. * indicates significant differences compared with the wild type (Student’s *t*-test, *P*≤0.05).

In order to study the role of INV and SUS in nematode development and offspring production, infection tests were performed on single and double mutants. T-DNA insertion lines of all six *SUS* genes and of the A/N-INV genes *CINV1* and *CINV2* that were studied most intensively, showing clear effects on plant development (Barratt *et al.*, 2009), were selected. The tests showed that *SUS* single mutants and the *Atcinv2* mutant did not have an effect on *H. schachtii* development or reproduction. In the *Atcinv1* mutant line, significantly more females developed (Supplementary Fig. S1 at *JXB* online). When two *SUS* genes were silenced simultaneously, positive effects on nematode development were observed. In the *Atsus1/Atsus4* and the *Atsus5/Atsus6* lines, female infections doubled compared with the wild type, and eggs per cyst rose to almost 150–200% (Fig. 3). Further, in the *Atsus1/Atsus4* line, female size was significantly increased. The *Atsus2/Atsus3* double mutant line presented an increase in total infection rate, while none of the developmental or reproductive aspects showed any significant deviation compared with the wild type. The double *Atcinv1/Atcinv2* mutant showed the most notable results (Fig. 3). Female development reached up to 400%, the total infection increased up to 300%, the females were 50% bigger, and the number of eggs per cyst was 120% higher than in the wild type. Thus, these data suggest that silencing *SUS* or *CINV* genes in double mutants is beneficial for nematode development. Further, the female to male ratio was calculated as a marker for *H. schachtii* living conditions. There is already evidence to show that sucrose addition into media of axenically cultivated plants enhanced female development, while the absence of sucrose promoted male nematode development (Grundler *et al.*, 1991). Especially in the *Atcinv1/Atcinv2* but also in the *Atsus1/Atsus4* line, the sex ratio significantly shifted towards the occurrence of females compared with the wild type (Fig. 4).

In order to study the results obtained from the infection assays in depth, detailed gene expression analyses were performed. The expression of *AtCINV1, AtCINV2*, and all six *SUS* genes was analysed in wild-type syncytia compared with non-infected control roots during nematode pathogenesis (5, 10, and 15 dai) (Fig. 3). At 5 dai, none of the studied genes was differentially expressed in syncytia compared with non-infected roots. *AtSUS1* and *AtSUS4* were significantly up-regulated at 10 dai, reaching the highest levels at 15 dai. Similarly, *AtSUS5* and *AtSUS6* expression increased significantly at 15 dai, but was steady between 5 and 10 dai. Fold change levels of *AtSUS2* and *AtSUS3* increased significantly at 10 dai (Fig. 3), but was not maintained over time. *AtCINV2* was not differentially expressed, but *AtCINV1* was significantly down-regulated at 10 and 15 dai. These data show that the *SUS* genes *AtSUS1* and *AtSUS4*, *AtSUS2* and *AtSUS3*, and *AtSUS5* and *AtSUS6* as well as *AtCINV1* and *AtCINV2* have a similar expression pattern during nematode development (Fig. 3). A Pearson’s correlation analysis for the aforementioned genes showed that *AtSUS1*, *AtSUS4*, and *AtSUS6* expression correlated strongly. Further, *AtSUS1* and *AtSUS4* showed the highest number of correlations. The *CINV* genes did not show any significant correlation (Table 2.)

**Table 2.** *Pearson’s correlation analysis of fold change (log*_*2*_ *) expression levels of* SUS *and* CINV *genes in* H. schachtii*-induced syncytia compared with non-infected* A. thaliana *roots 5, 10, and 15 dai*

	*AtCINV1*	*AtCINV2*	*AtSUS1*	*AtSUS2*	*AtSUS3*	*AtSUS4*	*AtSUS5*	*AtSUS6*
*AtCINV1*	1							
*AtCINV2*	0.507	1						
*AtSUS1*	0.122	0.297	1					
*AtSUS2*	0.441	0.401	**0.724**	1				
*AtSUS3*	0.189	0.593	0.392	0.615	1			
*AtSUS4*	0.203	0.493	**0.937**	**0.747**	0.449	1		
*AtSUS5*	0.294	0.158	**0.851**	**0.738**	0.497	**0.753**	1	
*AtSUS6*	0.195	0.307	**0.912**	0.678	0.426	**0.923**	**0.888**	1

Values in bold represent significant correlations (*P*≤0.05, Student’s *t*-test, –0.7 <*r*> 0.7). Note that the correlation coefficient *r* is displayed.

### Effects of *INV* and *SUS* mutation on sucrose processing in *H. schachtii*-infected roots

Since the *Atsus1/Atsus4* and *Atcinv1/Atcinv2* double mutants showed the greatest effects on nematode development and reproduction, sucrose breakdown in these mutants was studied in detail in nematode-infected plants. Enzyme activity assays revealed that the *Atcinv1/Atcinv2* double mutant showed no change in cytosolic, cell wall, or vacuolar INV activity in syncytia at 15 dai. Cytosolic INV activities were also not affected in syncytia of the *Atsus1/Atsus4* line, but vacuolar and cell wall INV activity was significantly increased (Fig. 1C–E). Thus, the total analysed INV activity was significantly increased in syncytia in the *Atsus1/Atsus4* line (Fig. 1F). Potential changes in sucrose synthase in the *Atcinv1/Atcinv2* line were studied transcriptionally by qPCR. None of the six *SUS* genes showed differential expression in the *Atcinv1/Atcinv2* double mutant compared with the wild type (Supplementary Fig. S2 at *JXB* online).

Finally, sugar levels of syncytia in the studied double mutants were analysed (Fig. 2A). As in the wild type, glucose, fructose, sucrose, and 1-kestose were the most abundant sugars. Syncytia in the *Atcinv1/Atcinv2* lines showed significantly increased sucrose, glucose, and fructose levels, while syncytia in the *Atsus1/Atsus4* line revealed increased trehalose and 1-kestose levels.

### *Heterodera schachtii* root infection affects systemic sucrose processing

Changes in syncytial sugar levels may indicate altered sink strength and thus changes in systemic sugar partitioning. Therefore, in the present study, sugar levels in shoots of nematode-infected plants were analysed (Supplementary Fig. S3 at *JXB* online). The most abundant sugars were, similarly to syncytia, glucose, fructose, and sucrose. In contrast to syncytia, in shoots nematode root infection triggered increased cytosolic, cell wall, and vacuolar INV activities (Fig. 1B). The *Atcinv1/Atcinv2* double mutant showed reduced CINV and increased CWINV activity compared with the control. The *Atsus1/Atsus4* double mutant had higher CWINV and VINV activity (Fig. 1C–E). This resulted in the highest INV activity levels in shoots and entire nematode-infected plants as compared with the other lines (Fig. 1F, G). However, changed enzyme activities were not reflected in altered sugar levels (Supplementary Fig. S3). In order to verify syncytial sink strength and sugar partitioning, the syncytia:i-shoot ratio of the single analysed sugars was calculated (Fig. 2B). The data revealed striking differences for 1-kestose and starch that were most enriched in syncytia or shoots, respectively (Fig. 2B). However, the syncytia:i-shoot ratios remained unaffected when comparing the *Atcinv1/Atcinv2* and the *Atsus1/Atsus4* double mutants with the wild type. In the *Atcinv1/Atcinv2* line, the sucrose, glucose, and fructose syncytia:i-shoot ratio was significantly changed.

### Effect on *Meloidogyne javanica*

**Fig. 5.**
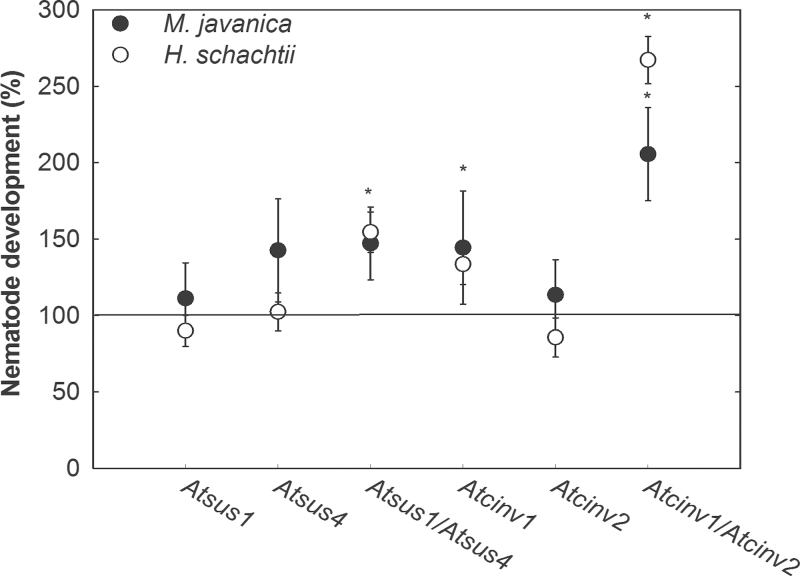
Nematode development in single and multiple *A. thaliana sus* and *cinv* T-DNA insertion lines compared with the wild type. To determine nematode development, for *H. schachtii* the number of females, and for *M. javanica* the number of galls was counted. Values are means±SE, *n*=10–18. * indicates significant differences compared with the wild type (Student’s *t*-test, *P*<0.05).

Next, it was examined whether the beneficial effects of lowered *SUS* and *INV* expression during development and reproduction of *H. schachtii* were specific for cyst nematodes or were a more common phenomenon during plant–nematode interactions. A previous transcript profiling showed that gene expression of *AtCINV2*, *AtSUS1*, and *AtSUS4* was up-regulated in 3-day-old galls and laser-microdissected giant cells of *M. javanica* (Barcala *et al.*, 2010) (Table 1). Thus, the role of sucrose degradation in the development of the root-knot nematode *M. javanica* was studied. The number of nematode-induced galls was assayed in the wild type as compared with the *Atsus4*, *Atsus1*, *Atsus1/Atsus4*, *Atcinv1*, *Atcinv2*, and *Atcinv1/Atcinv2* mutant lines. Figure 5 shows a significantly increased gall formation in the *Atsus1/Atsus4* and *Atcinv1* lines, and especially in the *Atcinv1/Atcinv2* lines. These increased nematode development rates closely match those observed for *H. schachtii*.

## Discussion

Pathogen-triggered changes in metabolite levels of their hosts may reflect a plant’s responses on the one hand and pathogen requirements on the other hand. In syncytial and giant cells, as well as for the feeding nematodes, sucrose cleavage by INV and SUS can be expected to play a significant role; however, to date, no studies had been performed.

### Sucrose cleaving efficiencies affect nematode development

In *A. thaliana*, 17 *INV* isoforms were described showing unique expression across plant organs and developmental stages (Vargas and Salerno, 2010); however, in NFS these genes were either down-regulated or not changed (Szakasits *et al.*, 2009; Barcala *et al.*, 2010). INVs are regulated at the post-transcriptional level by proteinase inhibitors, kinases, or compartmentalization (Rausch and Greiner, 2004; Huang *et al.*, 2007), so that transcriptional analyses do not necessarily demonstrate the real extent of enzymatic activity changes. In syncytia, enzyme activities of all three INV groups were reduced, which matched the reduced expression of *VINV1*, *CINV1*, *CWINV1*, and *CWINV6* which have been described as defective INVs (Le Roy *et al.*, 2013) (Table 1). This indicates that in syncytia these genes are predominantly responsible for INV activity and that they are transcriptionally regulated.

Silencing *AtCINV1* and *AtCINV2*, predicted to be most abundant in root cells (Barratt *et al.*, 2009), in a double mutant had beneficial effects for both of the studied nematode species (Fig. 5). Since the double mutation had no effect on syncytial INV activity compared with the wild type (Fig. 1), it can be suggested that other INV isoforms have become activated post-transcriptionally. This is supported by elevated glucose and fructose levels in syncytia of the mutant compared with syncytia of wild-type roots (Fig. 2A). The increased sucrose levels may be related to altered import efficiencies and sink–source relationships, as suggested before (Trouverie *et al.*, 2003). Elevated sugar pools were shown to contribute substantially to enhanced nematode development (Grundler *et al.*, 1991) and may thus have major nutritional value for the obligate parasites that may cleave sucrose with their own INVs. In agreement with this, INV activity has been described in *Pratylenchus penetrans*, *Panagrellus redivivus*, and *M. javanica* (Myers, 1965; Claussen and Bird, 1984), and in *M. incognita* two *INV* gene were identified (Abad *et al.*, 2008).

In addition to INVs, SUSs may play an essential role in sucrose cleavage in NFS. In plants, the different *SUS* isoforms show specific temporal and spatial expression patterns and have different roles during plant development and stress response (Angeles-Nunez and Tiessen, 2010). In contrast to *INV* genes, *SUS* genes were either up-regulated or not changed in NFS (Fig. 3; Szakasits *et al.*, 2009; Barcala *et al.*, 2010). Nematodes may favour SUS activity that is generating UDP-glucose. UDP-glucose can be directly used for starch, cell wall, and callose synthesis (reviewed by Koch, 2004), which were described to play significant roles during syncytium expansion and nematode development (Hofmann *et al.*, 2008, 2010b; Wieczorek *et al.*, 2008). In agreement with this, *AtSUS2* and *AtSUS3* were shown to be involved in starch synthesis (Baud *et al.*, 2004; Angeles-Nunez and Tiessen, 2010), and *AtSUS5* and *AtSUS6* were found to be associated with callose deposition in phloem elements (Barratt *et al.*, 2009).

Consistent with the results obtained for the *cinv1/cinv2* line, silencing *SUS* genes in double mutants had significantly beneficial effects on nematode development. Amongst these, the *Atsus1/Atsus4* line offered the best living conditions for both assayed nematode species. The two genes showed 95% similarity (Baud *et al.*, 2004) and exhibit overlapping but also distinct expression profiles. *AtSUS1* is expressed throughout the plant and *AtSUS4* is mainly confined to roots (Bieniawska *et al.*, 2007); both genes were up-regulated in syncytia (Fig. 3, Table 2).

### Invertases and sucrose synthases play particular roles in nematode-induced feeding sites

Even though INVs and SUS both cleave sucrose, the enzymes showed different involvement in the plant’s metabolism, although their impact has so far not been fully uncovered (Bieniawska *et al.*, 2007; Barratt *et al.*, 2009). In nematode-induced syncytia, transcripts of the enzymes are differentially regulated. INVs were described as post-transcriptionally regulated when plants face biotic stressors (Bonfig *et al.*, 2010); and while SUSs were also regulated on the transcriptional level, the induction of *AtSUS1* and *AtSUS4* gene expression has been reported during plant–pathogen interactions (Deeken *et al.*, 2006). Further, the *Atsus1/Atsus4* line presented no altered sucrose, glucose, and fructose levels in roots (Bieniawska *et al.*, 2007) and syncytia (this study). The observed increase in CWINV and VINV activity of nematode-infected *Atsus1/Atsus4* plants (Fig. 1D, E) may compensate for the lack of AtSUS4 that is localized not only in the cytosol but also in membranes and vacuoles (Szponarski *et al.*, 2004). The strongly reduced SUS activity in the *Atsus1/Atsus4* line (Bieniawska *et al.*, 2007) does not indicate that other SUS isoforms may have taken over sucrose cleavage. The increased sugar pools in the *Atsus1/Atsus4* line were trehalose and 1-kestose (Fig. 2A), which may enhance nematode development. Trehalose is a disaccharide that is synthesized by trehalose-6-phosphate synthase from UDP-glucose, a product of sucrose breakdown by SUS (Wingler, 2002). It accumulates under drought, high salinity, and cold stress (Wingler, 2002), it is fundamental for the infection by several pathogens (reviewed by Fernandez *et al.*, 2010), and is enriched during nematode infection (Hofmann *et al.*, 2010a). Even though its role is not yet fully understood, it has been proposed as a sugar signal (Lunn *et al.*, 2006). This may also apply to syncytia since trehalose levels were comparably low. Similarly to trehalose, 1-kestose accumulated during cold stress, stabilizing cell membranes in plant species such as chicory and Jerusalem artichoke (Livingston *et al.*, 2009). Even though 1-kestose enrichment during the *A. thaliana*–*H. schachtii* interaction was reported before (Hofmann *et al.*, 2010a), there is currently no information about its metabolism and its role in the non-fructan-accumulating *A. thaliana*. Fructan biosynthesis was shown to be stimulated by high sucrose and trehalose levels (Cairns and Ashton, 1991; Müller *et al.*, 2000; De Coninck *et al.*, 2005). In the current work its levels also correlated with trehalose, as well as with VINV activity in syncytia of the *Atsus1/Atsus4* line.

### Changes in *INV* and *SUS* expression may affect nematode-triggered plant stress response

The activity of INVs and SUSs may not only be responsible for providing fructose and glucose for the plant cell’s catabolism and anabolism, but were suggested to be involved in plant stress responses and signalling cascades (reviewed by Bolouri-Moghaddam *et al.*, 2010). In fact, the expression of the different INVs was found to be related to plant defence, PR-gene expression, antioxidative enzymes, and changing salicylic acid levels (Herbers *et al.*, 1996; Bonfig *et al.*, 2008, 2010; Bolouri-Moghaddam *et al.*, 2010). Further, changes in free hexose levels may act as signalling compounds regulating plant cell gene expression and activating Snf1-related kinase1, the kinase *AtPIP5K9*, and hexokinases (Koch, 1996; Tiessen *et al.*, 2003; Lou *et al.*, 2007). These kinases are also described as involved in hormone signalling and balancing reactive oxygen species (Koch, 1996, 2004; Rolland *et al.*, 2001; Camacho-Pereira *et al.*, 2009). The exact molecular mechanisms involved in sugar signalling are still largely unknown and thus also so are their role during plant–nematode interactions. In the study of Bieniewska *et al.* (2007), *SUS1* and *SUS4* were further shown to be up-regulated in roots facing hypoxia. Nematode-induced syncytia are described as metabolically highly active (Hofmann *et al.*, 2010a) which may increase oxygen demand and potentially lead to hypoxia. Transcriptome analyses showed that the alcohol dehydrogenase gene At1g77120, and At2G16060 coding for a non-symbiotic leghaemoglobin are significantly up-regulated in syncytia and in giant cells, indicating hypoxia (Szakasits *et al.*, 2009; Barcala *et al.*, 2010).

### The altered sink character of syncytia affects systemic sucrose processing

Sugars further act as systemic signalling compounds in plants, facilitating systemic communication between source and sink tissues. NFS have previously been described as metabolic sinks in the plant’s circulation system (Böckenhoff *et al.*, 1996; Hofmann *et al.*, 2007, 2010a; Baldacci-Cresp *et al.*, 2011). This was also observed in the present work showing that most sugar pools were elevated in syncytia; only starch levels were higher in the shoots. The data further indicate that the sink character of syncytia in the *Atcinv1/Atcinv2* double mutant was increased as, for example, the syncytia:shoot ratios of sucrose, glucose, and fructose, but also of 1-kestose were increased compared with the wild type (Fig. 2B). Changes in INV or SUS activity in source tissues may affect phloem loading and thus local and systemic sugar levels (Sonnewald *et al.*, 1991) as well as systemic sugar signalling (Koch, 1996, 2004). In agreement with this, silencing *Atcinv1/Atcinv2* or *Atsus1/Atsus4* triggered increased VINV and CWINV activity in shoots of nematode-infected plants. Further, INV and SUS may affect phloem unloading determining sink strength (Martin *et al.*, 1993; Koch, 1996; Ehness *et al.*, 1997; Fotopoulos *et al.*, 2003). Systemic changes in INV and SUS activity have frequently been described during plant–pathogen interactions. Root CWINV activity in *Vicia faba* was affected by the shoot pathogen *Uromyces fabae* (Voegele *et al.*, 2006). In *Solanum lycopersicum* (formerly *Lycopersicon esculentum*), the shoot pathogen *Botrytis cinerea* induced the transcription of two *CWINV* genes in fruits (Hyun *et al.*, 2011). VINV transcripts increased in shoots when roots were treated with hexanoyl homoserine lactones, a signal molecule of Gram-negative bacteria (Schuhegger *et al.*, 2006).

Summarizing, the present results revealed that SUS and INV play distinct and significant roles during plant–nematode interactions. This was reflected by different transcript and sugar levels that were beneficial for both tested nematode species. Changes in INV and SUS expression led to alterations in the delicate balance of local and systemic sugar processing and signalling. This affects the metabolism of the plant cells as well as systemic plant communication, source–sink relationships, and also nutrition of the parasitic nematodes.

## Supplementary data

Supplementary data are available at *JXB* online.

Figure S1. Expression of single *SUS* and *CINV* genes in syncytia of wild-type roots and impact of single *SUS* and *CINV* mutants in nematode development

Figure S2. Fold change of *SUS* gene expression in syncytia of the *Atcinv1/Atcinv2* line.

Figure S3. Sugar levels of shoots of nematode-infected plants.

Table S1. List of applied T-DNA lines.

Table S2. Primer sequence used to identified the correct T-DNA insertion in *SUS* and *CINV* genes.

Table S3. Primers sequences and concentrations, and MgCl_2_ concentrations for q-PCR

Supplementary Data
