# Supplementary material for: Altered sucrose synthase and invertase expression affects the local and systemic sugar metabolism of nematode-infected Arabidopsis thaliana plants
Source: J Exp Bot. 2013 Nov 1;65(1):201–12. doi: 10.1093/jxb/ert359 (PMC3883288; doi:10.1093/jxb/ert359)
Supplement: Supplementary Data [file supp_65_1_201__index.html]

Altered sucrose synthase and invertase expression affects the local and systemic sugar metabolism of nematode-infected Arabidopsis thaliana plants — Altered sucrose synthase and invertase expression affects the local and systemic sugar metabolism of nematode-infected Arabidopsis thaliana plants — Supplementary Data 

# Altered sucrose synthase and invertase expression affects the local and systemic sugar metabolism of nematode-infected *Arabidopsis thaliana* plants

## Supplementary Data

Data files

**Files in this Data Supplement:**

- Supplementary Data - Supplementary Data
