# Supplementary material for: Altered sucrose synthase and invertase expression affects the local and systemic sugar metabolism of nematode-infected Arabidopsis thaliana plants
Source: J Exp Bot. 2013 Nov 1;65(1):201–12. doi: 10.1093/jxb/ert359 (PMC3883288; doi:10.1093/jxb/ert359)
Supplement: Supplementary Data [file supp_ert359_jexbot106039_file001.pdf]

Altered sucrose synthase and invertase expression affects the local and systemic sugar metabolism of nematode-infected *Arabidopsis thaliana* plants

Susana Cabello[1], Cindy Lorenz[2], Sara Crespo[1], Javier Cabrera[3], Roland Ludwig[2], Carolina Escobar[3] & Julia Hofmann[1*]

Figure S1. Fold change (log_2) expression levels of *SUS* and *CINV* genes in *H. schachtii*-induced syncytia compared to non-infected *A. thaliana* wild type roots. Values are means ±SE, n=3 (left side). Total and female nematode infection rates, female size and eggs per cyst of *H. schachtii* on single *A. thaliana sus* and *cinv* T-DNA mutant lines relative to the wild type. Values are means ±SE, n=14 to 33 (right side). * indicate significant differences (Student's t-test, $p \leq 0.05$).

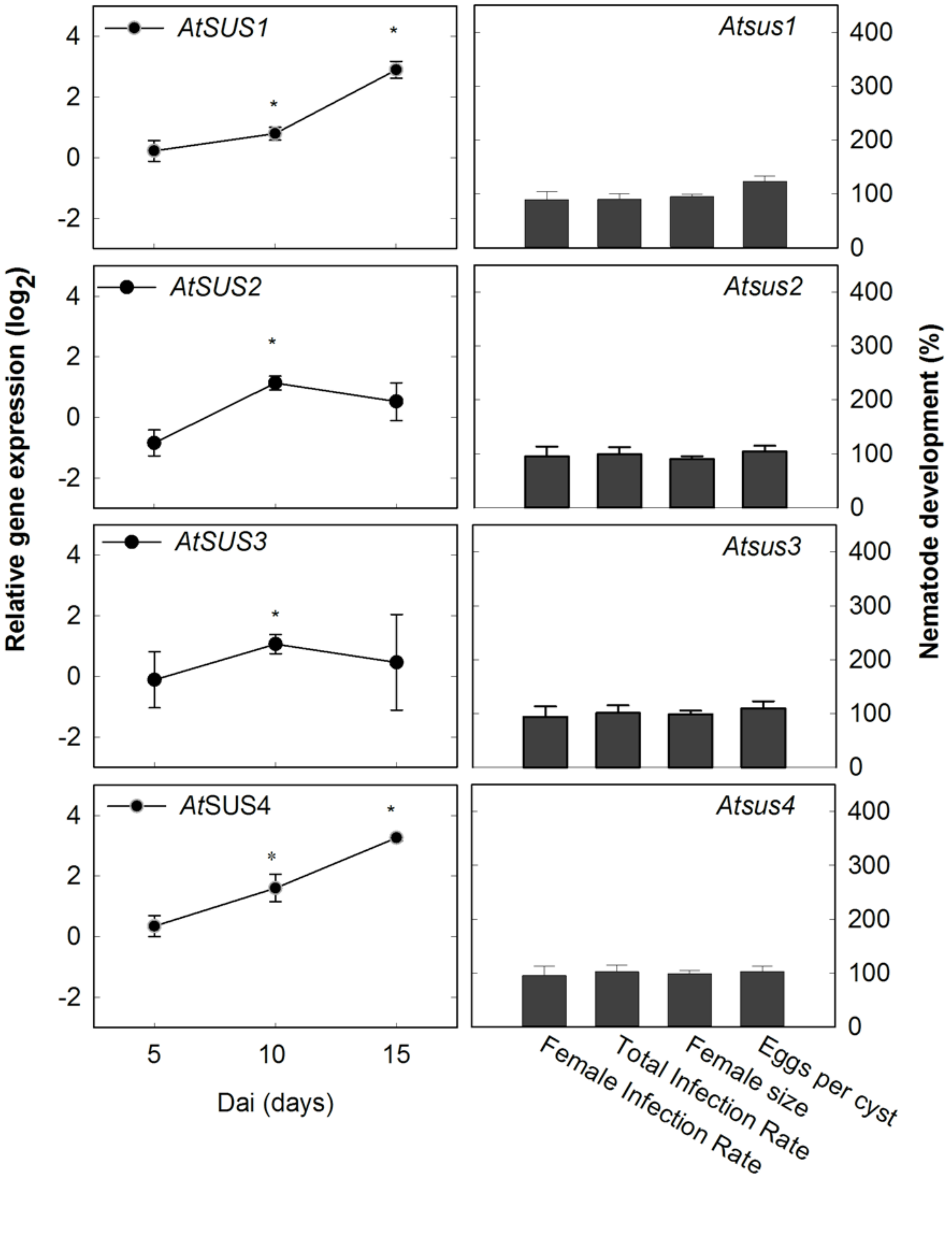

AtSUS1
AtSUS2
AtSUS3
AtSUS4
Atsus1
Atsus2
Atsus3
Atsus4
Relative gene expression (log2)
Nematode development (%)
Dai (days)
5
10
15
Female Infection Rate
Total Infection Rate
Female size
Eggs per cyst

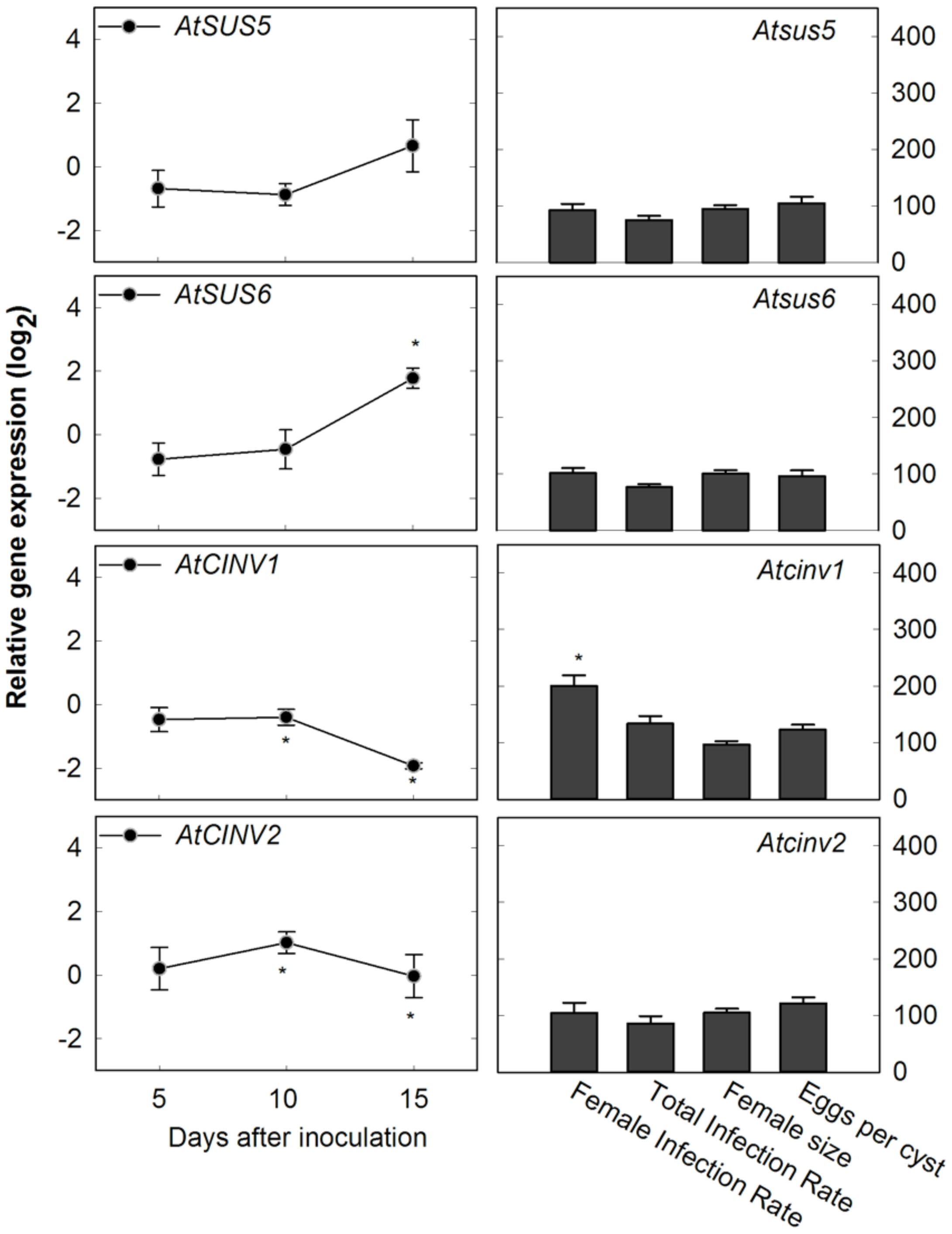

AtSUS5
Atsus5
AtSUS6
Atsus6
AtCINV1
Atcinv1
AtCINV2
Atcinv2
Relative gene expression (log_2)
Nematode development (%)
Days after inoculation
Female Infection Rate
Total Infection Rate
Female size
Eggs per cyst

Altered sucrose synthase and invertase expression affects the local and systemic sugar metabolism of nematode-infected *Arabidopsis thaliana* plants

Susana Cabello[1], Cindy Lorenz[2], Sara Crespo[1], Javier Cabrera[3], Roland Ludwig[2], Carolina Escobar[3] & Julia Hofmann[1*]

Figure S2. Fold change (log_2) expression levels of *SUS* genes in *H. schachtii*-induced syncytia of the *Atcinv1/Atcinv2* line compared to the wild type. Values are means ±SE, n=3.

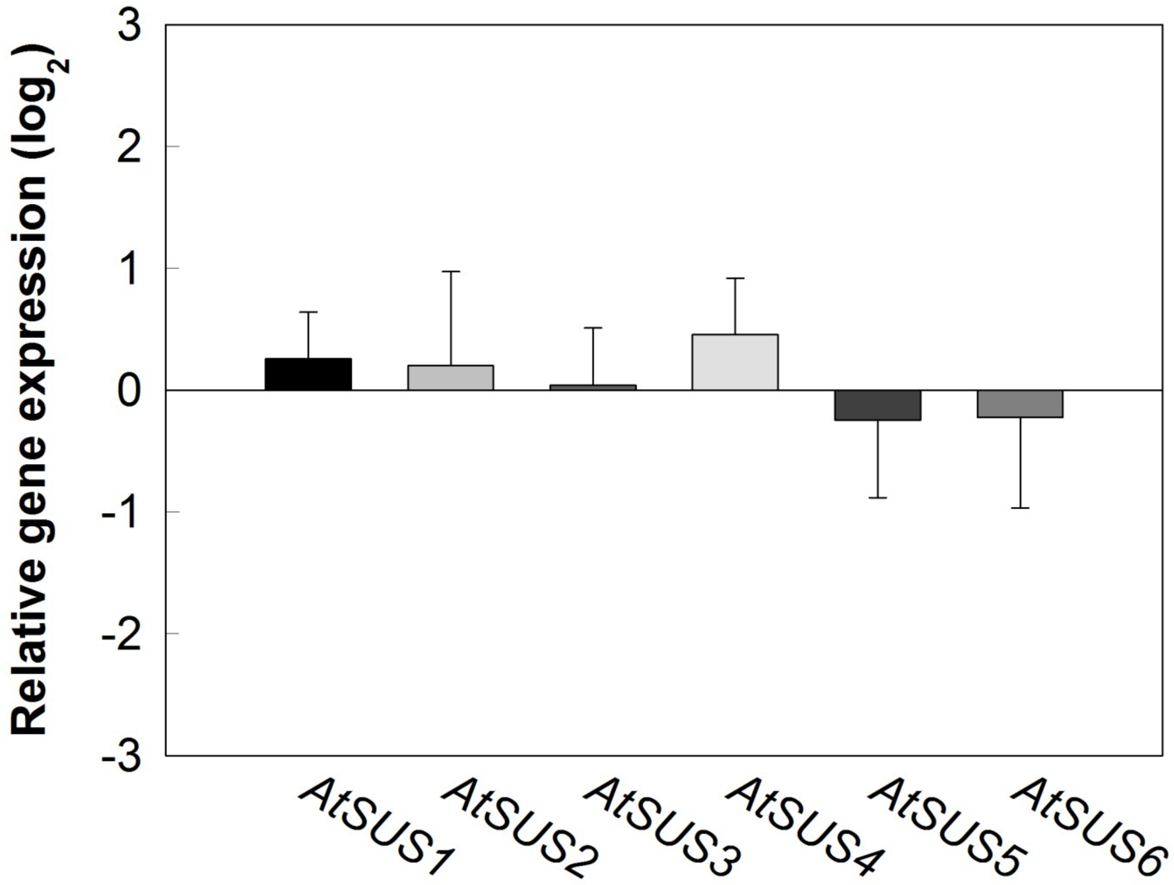

Altered sucrose synthase and invertase expression affects the local and systemic sugar metabolism of nematode-infected *Arabidopsis thaliana* plants

Susana Cabello[1], Cindy Lorenz[2], Sara Crespo[1], Javier Cabrera[3], Roland Ludwig[2], Carolina Escobar[3] & Julia Hofmann[1*]

Figure S3. Sugar levels of shoots of nematode-infected wild type, *cinv1/cinv2* and *sus1/sus4* T-DNA insertion lines (15 dai). Values are means ±SE, n=3, no significant differences from the wild type were observed (Student's t-test, p ≤ 0.05).

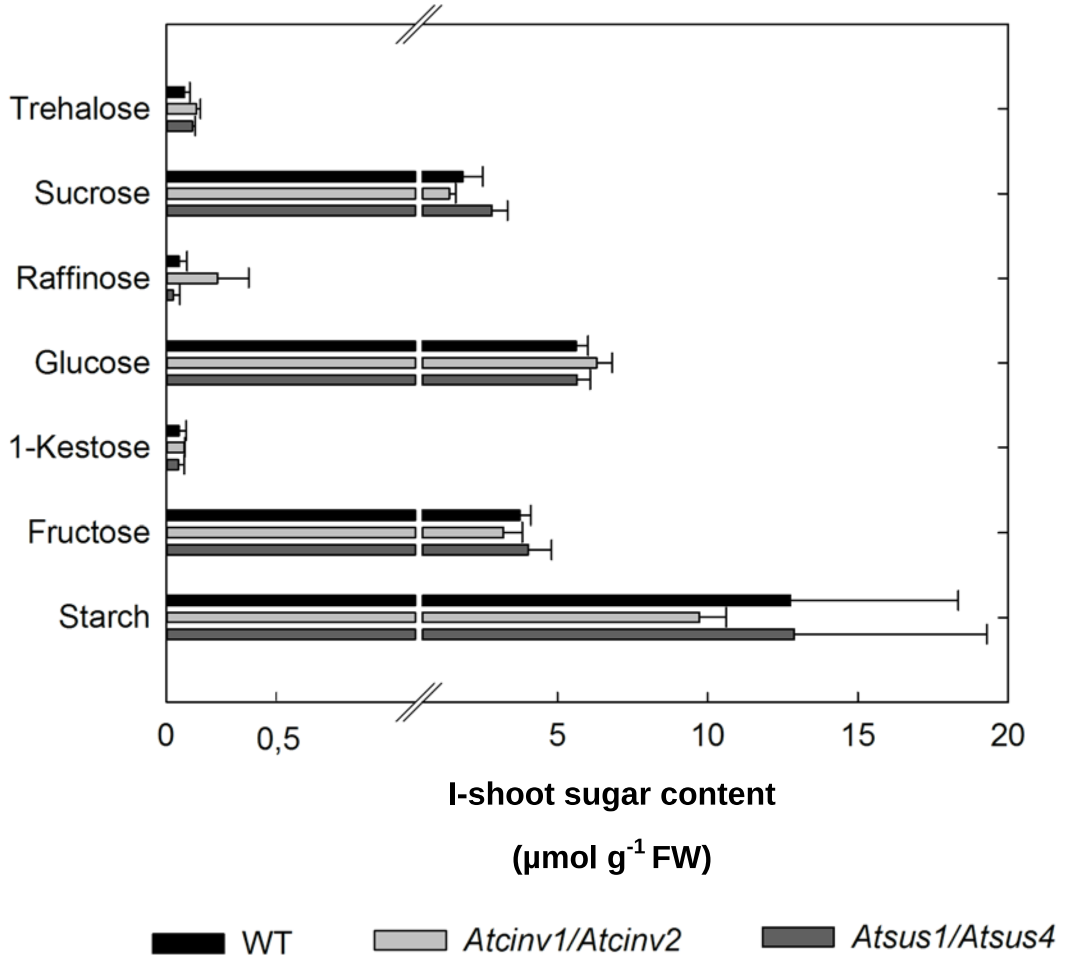

Altered sucrose synthase and invertase expression affects the local and systemic sugar metabolism of nematode-infected *Arabidopsis thaliana* plants

Susana Cabello[1], Cindy Lorenz[2], Sara Crespo[1], Javier Cabrera[3], Roland Ludwig[2], Carolina Escobar[3] & Julia Hofmann[1*]

Table S1: List of T-DNA insertion lines used in this study and their sources. All lines have the Arabidopsis Col0 background.

Name	Locus	Salk line	Source
Atsus1	At5g20830	SALK-014303	NASC
Atsus2	At5g49190	SALK-076303	NASC
Atsus3	At4g02280	SALK-019405	NASC
Atsus4	At3g43190	Arab4	Bieniawska et al., 2007
Atsus5	At1g73370	SAIL-374-D07	NASC
Atcinv1	At1g35580	Salk_095807	NASC
Atcinv2	At4g09510	Sail_518_d02	NASC
Atsus3/Atsus2	At4g02280	Salk_019405	Bieniawska et al., 2007
	At5g49190	Salk_076303	
Atsus5/Atsus6	At5g37180	Salk_152944	Bieniawska et al., 2007
	At1g73370	Salk_107491	
Atsus1/Atsus4	At4g20830	Salk_014303	Bieniawska et al., 2007
	At3g43190	Arab4	
Atcinv1/Atcinv2	At1g35580	Salk_095807	Barratt et al., 2009
	At4g09510	Sail_518_d02	

Altered sucrose synthase and invertase expression affects the local and systemic sugar metabolism of nematode-infected *Arabidopsis thaliana* plants

Susana Cabello[1], Cindy Lorenz[2], Sara Crespo[1], Javier Cabrera[3], Roland Ludwig[2], Carolina Escobar[3] & Julia Hofmann[1*]

Table S2. Primer sequence used to identified the correct T-DNA insertion in *SUS* and *CINV* genes

Gene	Primer sequence
At1g35580	RP CAATCGACCAAATGAGTGAGG
	LP CGCTAGACCTAGCCATTAGGG
At4g09510	RP AACGATCTCTTCCGTTCGATC
	LP GAGCCACATAATTCAAAGCAAC
At5g20830	RP CGCTTGTTTCTGAGAGAAACG
	LP TCCCCTCTCAAGACCAATCTC
At5g49190	RP TGCACCATTTCTGAAACCTT
	LP CATGGGTAATGGTTTTGGTTG
At4g02280	RP TTTCCCGTATTCCGAAGAAAC
	LP TTGGAGACCAGCGTCTGATAC
At5g37180	RP TTGTTTGGCCAGTTTCTGATC
	LP ATTTCCCTTTTACCGCACAAG
At1g73370	RP ATCCATCTGAATTTCCCCTTG
	LP TGACACGGTTAATACCGGAAG

Altered sucrose synthase and invertase expression affects the local and systemic sugar metabolism of nematode-infected *Arabidopsis thaliana* plants

Susana Cabello[1], Cindy Lorenz[2], Sara Crespo[1], Javier Cabrera[3], Roland Ludwig[2], Carolina Escobar[3] & Julia Hofmann[1*]

Table S3. Primers sequences, concentrations and $MgCl_2$ concentrations for q-PCR

**Gene**		**Primer**	**sequence**	**Primer (nM)**	**$MgCl_2$ (nM)**
At1g35580	*AtCINV1*	FP	TCGAGGGCCATGAGTGGCGC	300	
		RP	CGCGCGTCTTGCGATCTGCG		
At4g09510	*AtCINV2*	FP	CGGTGGATCTTGGCCAGTATTGC	200	
		RP	CCAGCAATCTCGGTGTAGCCGT		
At3g13790	*AtCWINV1*	FP	GCCAGCTCGTTCCGTGACCC	200	
		RP	ACGAAGACGTTTCCACGCCGTT		
At3g52600	*AtCWINV2*	FP	CGTGAAGGGTTGGGCCGGAA	200	
		RP	GGACCTCAAAGCGTTGGCCC		
At1g55120	*AtCWINV3*	FP	ATCTCAACCAACCGTACCGGACC	200	
		RP	CGTGGAGTGTCCCCACACGA		
At2g36190	*AtCWINV4*	FP	ACCAAAGGTGCGGTTTGGGGT	200	
		RP	TGAACCGGACCATGTACCTCCG		
At3g13784	*AtCWINV5*	FP	GGTTGGGCCGGTTTACAGGCT	150	
		RP	GGATCGGTCCAGCCCGGTTC		
At5g11920	*AtCWINV6*	FP	GCATCACTGCTGCCCAAGCA	200	25
		RP	TGCATATCCGTTACCGCGACGA		
At1g62660	*AtVACINV1*	FP	GGCGAGCACGGAAGCTCTCT	200	
		RP	TCAGCCTTGGAGCCGTCGTG		
At1g12240	*AtVACINV2*	FP	GTGGACCGGCTCAGCCACAT	200	
		RP	AGGGAGGATACCGGGCGGAG		
At5g20830	*AtSUS1*	FP	GCGCGTCCACAGCCAACGTG	200	
		RP	ACCAGGCCTTGGCCTCACAGC		
At5g49190	*AtSUS2*	FP	AGGGTGTACCAAATCTCAT	300	25
		RP	CATAGTGAAAGCTGTGTGG		
At4g02280	*AtSUS3*	FP	GAGCACGGGCTCTCGGGTTT	200	
		RP	GCCGAGTCTCACGACGCTCC		
At3g43190	*AtSUS4*	FP	CACACTTCCCGGGTTGTACCGT	350	25
		RP	GCGCAAGCGAGTGTTCTTACCG		
At5g37180	*AtSUS5*	FP	GTCCTCGAAGCTCGGAGGGC	180	
		RP	CTCCCTGCGCTTTCTCCCCA		
At1g73370	*AtSUS6*	FP	CGCCTTGATTGCAAGCCAGACC	200	
		RP	TGGCCTGTCCTTGCTTCCTGC		

At5g10790	*UBP22*	FP	ACAACATATGACCCGTTTATCGA	347	30
		RP	TGTTTAGGCGGAACGGATACT		
	18SrRNA	FP	GGTGGTAACGGGTGACGGAGAAT	355	30
		RP	CGCCGACCGAAGGGACAAGCCGA		
